# From Pure Contentment To Frustration: Older Pain Clinic Patients’ Experiences Of Close Relationships

**DOI:** 10.1093/geroni/igaf122.3374

**Published:** 2025-12-31

**Authors:** Meredith Stensland

**Affiliations:** The University of Texas Health Science Center at San Antonio, San Antonio, Texas, United States

## Abstract

Chronic low back pain (CLBP) is a prevalent condition among older adults, and it has profound physical, psychological, and social consequences. Older adults’ subjective experiences of close relationships while living with chronic pain have received limited attention, despite the important implications they have for how they manage their pain. Past research has been focused predominantly on younger and middle-aged adults, heavily quantitative, and reliant on statistical analyses that model support and strain as two siloed phenomena. Thus, to address these limitations, the purpose of the present study was to understand how older pain clinic patients subjectively experience close relationships and to examine co-existing interpersonal support and strain. Using van Manen’s phenomenological method, semi-structured, in-depth, one-on-one interviews were conducted with 21 older adults living with CLBP recruited from five outpatient pain clinics (aged 66–83 years). Data were iteratively analyzed via line-by-line thematic coding. Findings illustrate how the relationships of the participants were characterized by the co-occurrence of both positive and negative factors, leading to complex connections with their family and friends. Three core qualitative themes emerged, including: (1) Supported but misunderstood; (2) Self-censoring pain from others; (3) Good intentions, poor outcomes. This study contributes a vivid illustration of older adults’ relationship experiences in the context of CLBP. Findings highlight the importance of assessing the social context for individuals with chronic pain and implications for interdisciplinary pain management. Further research is needed to understand how coexisting support and strain impact pain and related psychosocial outcomes throughout the life course.

